# Xenografting of human umbilical mesenchymal stem cells from Wharton’s jelly ameliorates mouse spinocerebellar ataxia type 1

**DOI:** 10.1186/s40035-019-0166-8

**Published:** 2019-09-05

**Authors:** Pei-Jiun Tsai, Chang-Ching Yeh, Wan-Jhen Huang, Ming-Yuan Min, Tzu-Hao Huang, Tsui-Ling Ko, Pei-Yu Huang, Tien-Hua Chen, Sanford P. C. Hsu, Bing-Wen Soong, Yu-Show Fu

**Affiliations:** 1 Institute of Anatomy and Cell Biology, School of Medicine, National Yang-Ming University, Taipei, Taiwan, Republic of China; 2 Trauma Center, Department of Surgery, Veterans General Hospital, Taipei, Taiwan, Republic of China; 3 Department of Critical Care Medicine, Veterans General Hospital, Taipei, Taiwan, Republic of China; 4 Department of Obstetrics and Gynecology, Veterans General Hospital, Taipei, Taiwan, Republic of China; 5 Institute of Clinical Medicine, National Yang-Ming University, Taipei, Taiwan, Republic of China; 6 Department of Obstetrics and Gynecology, National Yang-Ming University, Taipei, Taiwan, Republic of China; 7 Department of Life Science, National Taiwan University, Taipei, Taiwan, Republic of China; 8 School of Medicine, I-Shou University, Kaohsiung, Taiwan, Republic of China; 9 Institute of Physiology, School of Medicine, National Yang-Ming University, Taipei, Taiwan, Republic of China; 10 Division of General Surgery, Department of Surgery, Veterans General Hospital, Taipei, Taiwan, Republic of China; 11 Department of Neurosurgery, Neurological Institute, Taipei Veterans General Hospital, Taipei, Taiwan, Republic of China; 12 School of Medicine, National Yang-Ming University, Taipei, Taiwan, Republic of China; 13 Department of Neurology, Shuang Ho Hospital, and Taipei Neuroscience Institute, Taipei Medical University, Taipei, Taiwan, Republic of China; 14 Department of Neurology, Taipei Veterans General Hospital, Taipei, Taiwan, Republic of China; 15 Brain Research Center, National Yang-Ming University, Taipei, Taiwan, Republic of China; 16 Department of Anatomy and Cell Biology, School of Medicine, National Yang-Ming University, No. 155, Sec. 2, Li-Nung Street, Taipei, 112 Taiwan, Republic of China

**Keywords:** Umbilical mesenchymal stem cells, Cell transplantation, SCA1

## Abstract

**Background:**

Spinocerebellar ataxia type 1 (SCA1) is an autosomal dominant neurodegenerative disorder caused by the expansion of CAG repeats in *ATXN1* gene resulting in an expansion of polyglutamine repeats in the ATXN1 protein. Unfortunately, there has yet been any effective treatment so far for SCA1. This study investigated the feasibility of transplanting human umbilical mesenchymal stem cells (HUMSCs) into transgenic SCA1 mice containing an expanded uninterrupted allele with 82 repeats in the *ATXN1-*coding region.

**Methods:**

10^6^ human umbilical mesenchymal stem cells were transplanted into the cerebella at 1 month of age.

**Results:**

HUMSCs displayed significant ameliorating effects in SCA1 mice in terms of motor behaviors in balance beam test and open field test as compared with the untransplanted SCA1 mice. HUMSCs transplantation effectively reduced the cerebellar atrophy, salvaged Purkinje cell death, and alleviated molecular layer shrinkage. Electrophysiological studies showed higher amplitudes of compound motor action potentials as indicated by increasing neuronal-muscular response strength to stimuli after stem cell transplantation. At 5 months after transplantation, HUMSCs scattering in the mice cerebella remained viable and secreted cytokines without differentiating into neuronal or glia cells.

**Conclusions:**

Our findings provide hope for a new therapeutic direction for the treatment of SCA1.

**Electronic supplementary material:**

The online version of this article (10.1186/s40035-019-0166-8) contains supplementary material, which is available to authorized users.

## Background

Spinocerebellar ataxia (SCA) is a group of autosomal dominantly inherited neurological disorders that are clinically and genetically heterogeneous. They are characterized by progressive gait unsteadiness, clumsiness of the hands, dysphagia and dysarthria [1, 2]. The abnormal expansion of a CAG-repeat encoding a poly-glutamine (polyQ) tract in *ATXN1* gene causes the disease spinocerebellar ataxia type 1 (SCA1) [3–7]. Pathologically, the disease is characterized by a loss of cerebellar Purkinje cells and neurons in the brainstem, fibers in the spinocerebellar tracts [8–10]. Currently, there is no effective treatment for SCA1. Studies have suggested that stem cell transplantation might potentially be able to repair the neurodegenerative disease [11–13].

Human mesenchymal cells from Wharton’s jelly of the umbilical cord are obtained from medical waste after delivery and therefore carry little ethical concerns. These human umbilical mesenchymal stem cells (HUMSCs) possess stem cell properties and are capable of differentiating into neurogenic, osteogenic, chondrogenic, adipogenic, and myogenic cells in vitro [14–16]. We previously have shown that HUMSCs are viable after being engrafted into the striatum, hippocampi, cerebral cortex and spinal cord of rats without the need for immunological suppression [17–21]. In addition to the central nervous system disorders, HUMSCs transplants also exhibit promising therapeutic potentials in rats with liver fibrosis, peritoneal fibrosis [22, 23] and type 1 diabetes [24]. These results indicate that HUMSCs possess the ability for long-term survival and remain functional within various host organs of the rat, suggesting that HUMSCs are a good stem cell source for xeno-transplantation.

In the present study, HUMSCs were isolated from Wharton’s jelly of human umbilical cords and transplanted into the cerebella of transgenic mice bearing SCA1 to investigate their possible therapeutic effects. The results showed that the transplanted cells remained viable and released cytokines for several months, effectively ameliorated motor and behavioral deficits and alleviated cerebellar atrophy and cell deaths in SCA1 transgenic mice.

## Materials and methods

### Experimental animals

**Fig. 1:**
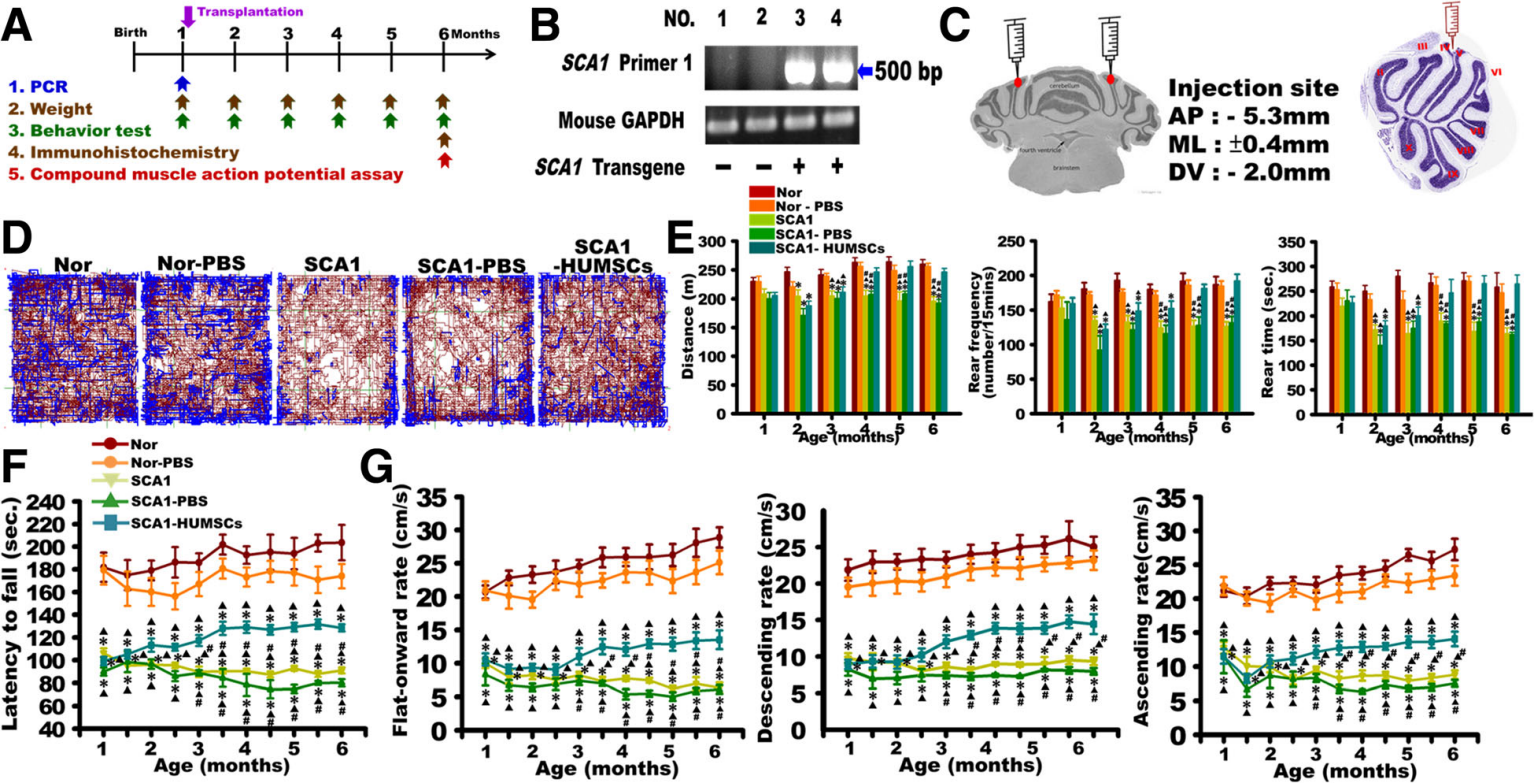
HUMSCs transplantation ameliorated motor behavior deterioration in SCA1 mice. **a** Scheme of experimental procedures. **b** Genotyping of SCA1 transgene. SCA1 Primer 1 set amplified a 500-bp amplicon. Lanes 3 and 4 show the presence of the SCA1 transgene. **c** The sketch and HE-stained picture show the injection site of HUMSCs. Open field tests revealed that HUMSCs transplants improved total distance traveled, rearing frequency, and rearing time in the SCA1-HUMSCs group mice (**d** and **e**). The group of SCA1-HUMSCs received, at 1 month of age, the HUMSCs transplantation which ameliorated the significant shortening of the latency to fall in the rotarod test (**f**), flat-forward, descending, and ascending speeds in the balance beam test (**g**) suffered by the SCA1 mice. * *p* < 0.05 compared with the Normal group of the same age. ▲ *p* < 0.05 compared with the Normal-PBS group of the same age. # *p* < 0.05 compared with the SCA1-HUMSCs group of the same age

SCA1 transgenic mice (B05 line) were kindly provided by Professor Harry Orr. The transgenic B05/+ line carrying a mutant SCA1 allele with 82 CAG repeats was established by Burright et al. [5]. The off-springs of parental strain PS-82B05 crossed with FVB mice were provided by National Laboratory Animal Center (Taipei, Taiwan). B05 homozygous mice were maintained and used in the experiments. The timeframe for various experiments is illustrated in Fig. 1a.

### Genotyping of experimental animals

Genotyping was performed by polymerase chain reaction (PCR). Genomic DNA was extracted from 1-cm tail snips of one-month-old mice and analyzed using a DNA mini kit (PCR reagent, Q-Amp™ 2x HotStart PCR Master Mix, Bio-Genesis, GL-HSMM-001). The primers were used to detect the abnormal CAG repeats. The primers 5EX2B (5′-AGGTTCACCGGACCAGGAAGG-3′) and RUBY (5′-AATGAACTGGAAGGTGTGCGGC-3′) could detect the abnormal 82-CAG triplet repeats within *ATXN1* which resulted in an amplicon of 500 bp. The PCR conditions were as follows: 35 cycles of 95 °C 1 min, 60 °C 30 s, and 72 °C 1 min (PCR machine, Select BioProduct, SelectCyclerII Thermal Cycler SBT9600). The PCR products were analyzed via (2%) agarose gel electrophoresis and visualized with UV translluminator. As shown in Fig. 1b, the 500 bp amplicon was detected in mice No. 3 and 4, indicating that they were SCA1 transgenic mice.

### Animal grouping

Animals were divided into five groups as follows:

1. The Normal group: Wild-type mice which did not carry the mutant human *ATXN1* transgene, *n* = 18.
2. The Normal-PBS group: Wild-type mice that received the same injection volume of the vehicle phosphate buffered saline (PBS), instead of stem cell transplants, into the cerebellum, *n* = 18.
3. The SCA1 group: Transgenic mice carrying the human *ATXN1* containing 82 CAG repeats. No surgery was performed, *n* = 12.
4. The SCA1-PBS group: Transgenic mice that carried the human *ATXN1* containing 82 CAG repeats received the same injection volume of the vehicle PBS, instead of stem cell transplants, into their cerebella 1 month after birth, *n* = 8.
5. The SCA1-HUMSCs group: Transgenic mice that carried the human *ATXN1* containing 82 CAG repeats and received HUMSCs transplants 1 month after birth, *n* = 15.

The weights and behaviors of these five groups were documented 1 month after birth and continued monthly until they were sacrificed at 6 months of age. The time-line of the experiments is shown in Fig. 1a.

#### Isolation and culture of HUMSCs

Human umbilical cords were collected aseptically after delivery and kept at 4 °C in Hank’s Balanced Salt Solution. The HUMSCs were isolated within 24 h after collection. All equipment was autoclaved before the experiments. During the experiment, all instruments were disinfected in 75% ethanol and flamed before reuse. In a laminar hood, the umbilical cords were disinfected in 75% ethanol and placed in the HBSS solution. Subsequently, the mesenchymal tissue (Wharton’s jelly) was cut into small pieces and centrifuged at 4000 rpm for 5 min. After removing the supernatant fraction, the umbilical mesenchymal tissue was treated with collagenase and trypsin, followed by the addition of fetal bovine serum (FBS; Gibco 10,437–028) to stop the reaction; at that point, the umbilical mesenchymal cells were fully processed into HUMSCs. Finally, the HUMSCs were suspended in 10% FBS Dulbecco’s modified Eagle’s medium (DMEM) for calculating cell number. The mesenchymal cells were then used directly for cultures or stored in liquid nitrogen for later use.

We previously demonstrated that similarly processed HUMSCs expressed high levels of matrix receptors (CD44 and CD105), integrin (CD29 and CD51), and mesenchymal stem cell markers (SH2 and SH3) but did not express hematopoietic lineage markers (CD34, CD45), suggesting that HUMSCs are similar to bone marrow mesenchymal stem cells [16] and Additional file 1: Figure S1B. The HUMSCs were mesenchymal-like in shape with a flat and polygonal morphology after treatment with 10% FBS DMEM for 3 days. With a continuous culture for 6 days, the HUMSCs were mostly a spindle shape and were closely attached to each other after proliferation [15]. These HUMSCs are capable of differentiating into neurogenic, osteogenic, chondrogenic, adipogenic, and cardiomyogenic cells in vitro using different differentiation media [15, 16]. HUMSCs did not display any chromosomal abnormality in the karyotype of HUMSCs in vitro using CytoScan 750 K Array (Affymetrix) (Additional file 1: Figure S1A).

The HUMSCs were treated with 0.05% Trypsin-EDTA (Gibco 15,400–054) for 2.5 min. Cells were then collected, washed twice with 10% FBS DMEM, and centrifuged at 1500 rpm for 5 min; then, the supernatant was removed. The pelleted cells were subsequently suspended at a concentration of 5 × 10^5^ in 10 μL of 0.1 M sterile PBS.

### Transplantation of HUMSCs

Under anesthesia (Zoletil®50, Sigma, XR-LZOL001-1EA, dose: 0.1 mL/100 g), 5 × 10^5^ HUMSCs were injected into both the left and right cerebellar cortex around the top of Lobules IV (AP:-5.3 mm, R/L:± 0.4 mm, H:-2.0 mm; total cell number: 1 × 10^6^ cells) of one-month-old transgenic mice (Fig. 1c). In the Normal-PBS and SCA1-PBS groups, 10 μl of PBS, instead of HUMSCs, was injected.

### Tracing of HUMSCs using bisbenzamide treatment and anti-human nuclear Ag immunohistochemistry

To trace the viability and distribution of the implanted HUMSCs, the cells were labeled with 1 g/ml bisbenzamide (Sigma B2883) for 48 h before trypsination and transplantation. Following sacrifice, mice were perfused with the fixative of 4% paraformaldhyde (Merck Un2213) and 7.5% picric acid (Sigma 925–40) in 0.1 M phosphate buffer (PB). The brain tissues were post-fixed in the same fixative for 24 h and cryoprotected in 30% sucrose in 0.1 M PB. Following sedimentation, the tissue was embedded in optimum cutting temperature compound, frozen, and cryo-sectioned at 30 μm in a cryostat (− 20 °C). Frozen sections were thaw-mounted onto coated glass slides, and directly observed and photographed under fluorescence microscope. In addition, immunostaining using anti-human specific nuclear antigen was performed to trace the survival of HUMSCs. Tissue sections were first reacted with a primary antibody (mouse anti-human-specific nuclear antigen, 1:100, Chemicon MAB1281, 1:100) at 4 °C for 18 h, washed with 0.1 M PBS, reacted with secondary antibodies (biotin-conjugated goat anti-mouse-IgG, 1:300 diluted, Sigma) at room temperature for 1 h. After the chromogenic reaction, the sections were coverslipped and observed under a microscope.

### Behavioral assessments

#### Rotarod test

A rotarod test was used to evaluate the coordinated movements of the limbs by measuring the latency at which the mice remained on a slowly accelerated spinning rod (increasing from 4 rpm to 40 rpm, Rotarod, SINGA, RT-01). The time for which experimental animals could stay on the rod before falling off was documented [25].

#### Balance beam test

Mice were placed on a round acrylic beam, 1.5-cm in diameter and 60 cm in length. The distance and time that the mice traversed the horizontal or 30° descending/ascending balance beam were recorded. The distance was divided by time to obtain the descending/ascending and flat-onward speeds [26].

#### Open field test

Six-month-old mice were placed in an arena of diameter 60 cm. The total distance travelled, rearing frequency, and rearing time were quantified during a 15- min period using the EthoVision software [27].

### Neuronal -muscular electrophysiology

The electrophysiological studies were conducted in 6-month-old mice. The stimulating electrodes were inserted into the muscles around the fourth lumbar vertebra and positioned on the spinal column to give 2 mA stimuli. The recording electrode was inserted into the gastrocnemius muscles to record the compound motor action potentials [28]. The latency, amplitude, and duration were thus measured (Electromyography machine, iWorx, IX/228S Data Acquisition System).

### Immunohistochemical staining of cerebella sections

Six-month-old mice were first sedated and then perfused with 4% paraformadehyde (Sigma 10,060) and 7.5% picric acid (Sigma 925–40). Serial sagittal sections of paraffin-embedded cerebella (10 μm thick) were used to examine the pathological changes. Successive sections as one group were processed in following sequence: Cresyl violet staining, anti-calbindin (1:1000, Chemicon), anti- ATXN1 protein (1:100, Cell Signaling 2177) and anti-human specific nuclei antigen (1:100, Millipore MAB1281) immunohistochemical staining. Middle cerebellar sections that showed more lobules and greater integrity were chosen for quantification. The cerebellar volume diminished in the groups of SCA1 and SCA1-PBS, and the total number of Purkinje cells in the cerebellum also declined in these two groups. To normalize the factor of cerebellar volume, the number of Purkinje cell in the unit length of Purkinje cell layer in the Lobules III or VI was shown for the quantification of the number of Purkinje cell (Additional file 2: Figure S2).

### Cresyl violet staining

Cresyl violet stains the Nissl bodies within neurons and therefore is commonly used to identify the neuronal structure. After deparaffinization and rehydration, the sections were soaked in 1% Cresyl violet solution for 20 min, followed by dehydration through immersion in a series of increasing alcohol concentrations (50, 70, 80, 90, 95, and 100%) and 100% xylene. Finally, slides were mounted with mounting media (Fisher Scientific SP15–500) and photographed under optic microscope. The widths and areas of molecular layer in lobules III and VI of six-month-old mice were quantified.

### Intracellular Biocytin injection and histochemistry for the morphology of Purkinje cells dendrites

Fresh mice cerebella were divided into right and left cerebellum. Sagittal brain slices were further sectioned at 300 μm. 1% biocytin (sigma B4261) in 2 M KCl was ionotophoretically injected into Purkinje cells by using 0.1–0.3 nA of depolarizing current for about 30 min at alternating intervals of 20 ms on and 20 ms off [29]. Brain slices were then fixed with 4% paraformaldehyde in 0.1 M PB at 4 °C for 16–18 h. Using a sliding microtome, serial frozen sections of 50 μm thickness were prepared and incubated in 0.5% H_2_O_2_ for 20 min to remove endogenous peroxidase. They were further blocked with blocking solution (3% Bovine serum albumin, 1% Triton X-100, 5% FBS) for 90 min at room temperature. Finally, slices were reacted with Avidin-biotinylated complex (ABC Kit, Vector) for 1 day, and developed with 3,3-diaminobenzidine. Focus was fine-tuned and all details of the cells were then depicted. Subsequently, the soma of the Purkinje cell was used as the center to quantify the maximal sagittal extension of their dendrites. Sholl analysis was applied to investigate the dendritic branches. Centered at the soma of the Purkinje cell, concentric circles with gradually increasing radii of 10 μm formed the grid. The numbers of ring-crossings of each branch (number of intersections) were counted and represented the complexity of dendritic branches [30].

### RT-PCR for detecting human NeuN and GFAP in the cerebellum of SCA1 mouse

Total RNA was freshly isolated from the mice cerebella with Tri Reagent (Q-Amp™ 2x HotStart PCR Master Mix, Bio-Genesis, GL-HSMM-001), reversely transcribed into complementary DNA with oligo (dT) primer, and amplified by 35 cycles (94 °C, 1 min; 55 °C, 1 min; and 72 °C, 1 min) of PCR with 10 pmol of specific primers. On completion of the PCR cycles, products were examined on 2% agarose gel. The mouse glyceraldehyde 3-phosphate dehydrogenase (GAPDH) primer was used as an internal standard. Human glioma cells were used as a positive control. The primer sequences were as follows:

- Mouse GAPDH
  - Sense: 5′-CCGGAGAATGGGAAGC-3′
  - Antisense: 5′-GTAGACGGTCTTGGGC-3′
- Human NeuN
  - Sense: 5′-ATCCAGTGGTCGGCGCAGTCTAC-3′
  - Antisense: 5′-TACGGGTCGGCAGCTGCGTA-3′
- Human glial fibrillary acidic protein (GFAP)
  - Sense: 5′-TCCACTTCCTCCTCCTCCACGA-3′
  - Antisense: 5′-AACTTGCACACGGCGCAGGT-3′

### Human cytokine array

To elucidate which human cytokines were involved in the protection of SCA1 cerebellum, a human protein cytokine kit (AAH-CYT-2000, RayBio Human Cytokine Antibody Array C Series 2000, RayBiotech) was used to screen the expression of 174 human cytokines (*n* = 3/group). The mice were deeply anesthetized and decapitated at 5 months after HUMSC transplantation. Cerebella were homogenized in lysis buffer and centrifuged at 1500 g to separate cell debris. The supernatant was harvested and then incubated for 2 h at room temperature with membranes containing an array of human cytokine antibodies. The levels of cytokine expression were determined by the intensities of immunoreactivity as relative to that of the standard controls using enhanced chemiluminescence according to the manufacturer’s instructions.

### Statistical analyses

All data were presented as means ± SEM (standard errors of the mean). One-way ANOVA was used to compare all means and Tukey’s test was applied for multiple comparisons. A value of *p*<0.05 was considered statistically significant.

## Results

### HUMSC transplantation ameliorated SCA1-induced motor behavior deterioration

Behavior patterns were compared by the open field test in which the total distance traveled within 15 min, rearing frequencies and total rearing time were quantified. The results indicated that the SCA1 and SCA1-PBS mice traveled shorter distances, exhibited lower rearing frequencies, and registered shorter total rearing time as compared to those in the Normal and Normal-PBS groups from two-months to six-months of age. HUMSCs transplantation resulted in significant improvements in all of these behavioral parameters starting from four-months of age (Fig. 1d and e).

Starting at one-month age, all mice in the other groups exhibited significant deterioration of motor coordination compared to those in the Normal and Normal-PBS groups as reflected in significantly shorter latencies to fall. However, the latencies in the SCA1-HUMSCs mice showed significant improvements as compared to those in the SCA1 and SCA1-PBS groups (Fig. 1f).

Motor performances, as assessed with the walking speed on flat forward and 30°ascending or descending balance beams showed similar trends. Average speeds were between 20.67 ± 1.00 to 28.88 ± 1.53 cm/sec for going flat-onward, between 20.02 ± 1.02 to 27.26 ± 1.59 for ascending and between 20.02 ± 1.70 to 26.15 ± 2.45 cm/sec for descending in the Normal and Normal-PBS groups (Fig. 1g). At 1 month of age, significantly lower speeds were found in the SCA1, SCA1-PBS, and SCA1-HUMSCs groups. When the SCA1 and SCA1-PBS mice grew older, the behavior got worse. Their hind-limbs often slipped from the beams when crossing the beams, and some mice held on the beams using their limbs, belly-crawling forward on the beams instead of running. The motor performance of the SCA1-HUMSCs group remained stable, indicating that transplantation of HUMSCs attenuated behavioral deterioration (Fig. 1g) (Additional file 5: Video S1, Additional file 6: Video S2, Additional file 7: Video S3).


**Additional file 5: Video S1.** The record of the flat-onward balance beam of the mice in the Normal, Normal-PBS, SCA1, SCA1-PBS, and SCA1-HUMSCs groups at 6 months of age. (MP4 1422 kb)



**Additional file 6: Video S2.** The record of the ascending balance beam of the mice in the Normal, Normal-PBS, SCA1, SCA1-PBS, and SCA1-HUMSCs groups at 6 months of age. (MP4 1528 kb)



**Additional file 7: Video S3.** The record of the descending balance beam of the mice in the Normal, Normal-PBS, SCA1, SCA1-PBS, and SCA1-HUMSCs groups at 6 months of age. (MP4 1559 kb)


### HUMSC transplantation attenuated SCA1-induced deterioration in limb muscle contractions

**Fig. 2:**
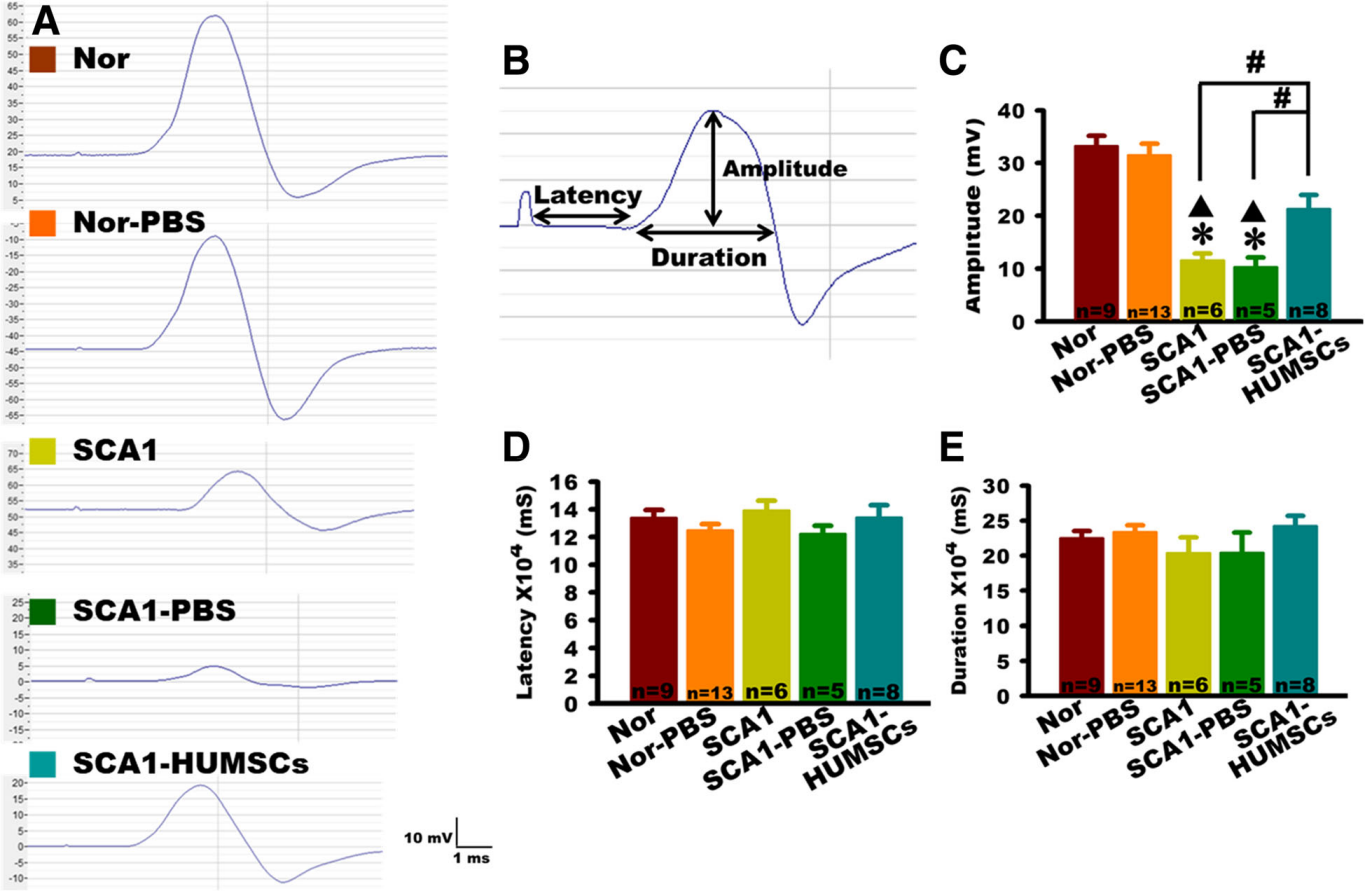
HUMSCs transplantation improved the amplitude of the compound motor action potentials in SCA1 mice. **a** Representative recordings of compound motor action potentials generated by the gastrocnemius muscles at 6 months of age for Normal, Normal-PBS, SCA1, SCA1-PBS, and SCA1-HUMSCs mice, showing markedly depressed action potentials in the SCA1 and SCA1-PBS groups and partial recovery by HUMSC transplantation. **b** Parameters of a schematic compound motor action potential assessed in the study. Quantifications of the mean amplitude, latency, and duration of the compound motor action potentials indicated, in the 6 months old SCA1 and SCA1-PBS mice, significant amplitude depressions which were partially recovered in the SCA1-HUMSCs group (**c**). However, no statistical differences were found among all the groups with respect to latency (**d**) and duration (**e**) recorded. * *p* < 0.05 compared with the Normal group. ▲ *p* < 0.05 compared with the Normal-PBS group. # *p* < 0.05 compared with the SCA1-HUMSCs group

Muscle contraction responses were assessed by electrophysiological analyses. Marked reductions of compound motor action potentials in the SCA1 and SCA1-PBS group mice, including significantly smaller amplitudes as measured from the baseline to the positive peak, indicated weakened muscular responses to neural stimuli. HUMSCs transplantation significantly improved such responses (Fig. 2a-c). However, no statistical differences were found between groups with respect to the latency and duration, representing respectively the nerve conduction time from the electrical stimulations of the lumbar roots to the beginning of the gastrocnemius muscle contraction and the period from the beginning of the gastrocnemius muscle contraction to the relaxation (Fig. 2b, d-e). Analyses of the electrophysiological results indicated that transplantation of HUMSCs preserved the number of neuronal innervation to gastrocnemius muscle.

### HUMSCs transplantation alleviated SCA1-induced cerebellar atrophy

**Fig. 3:**
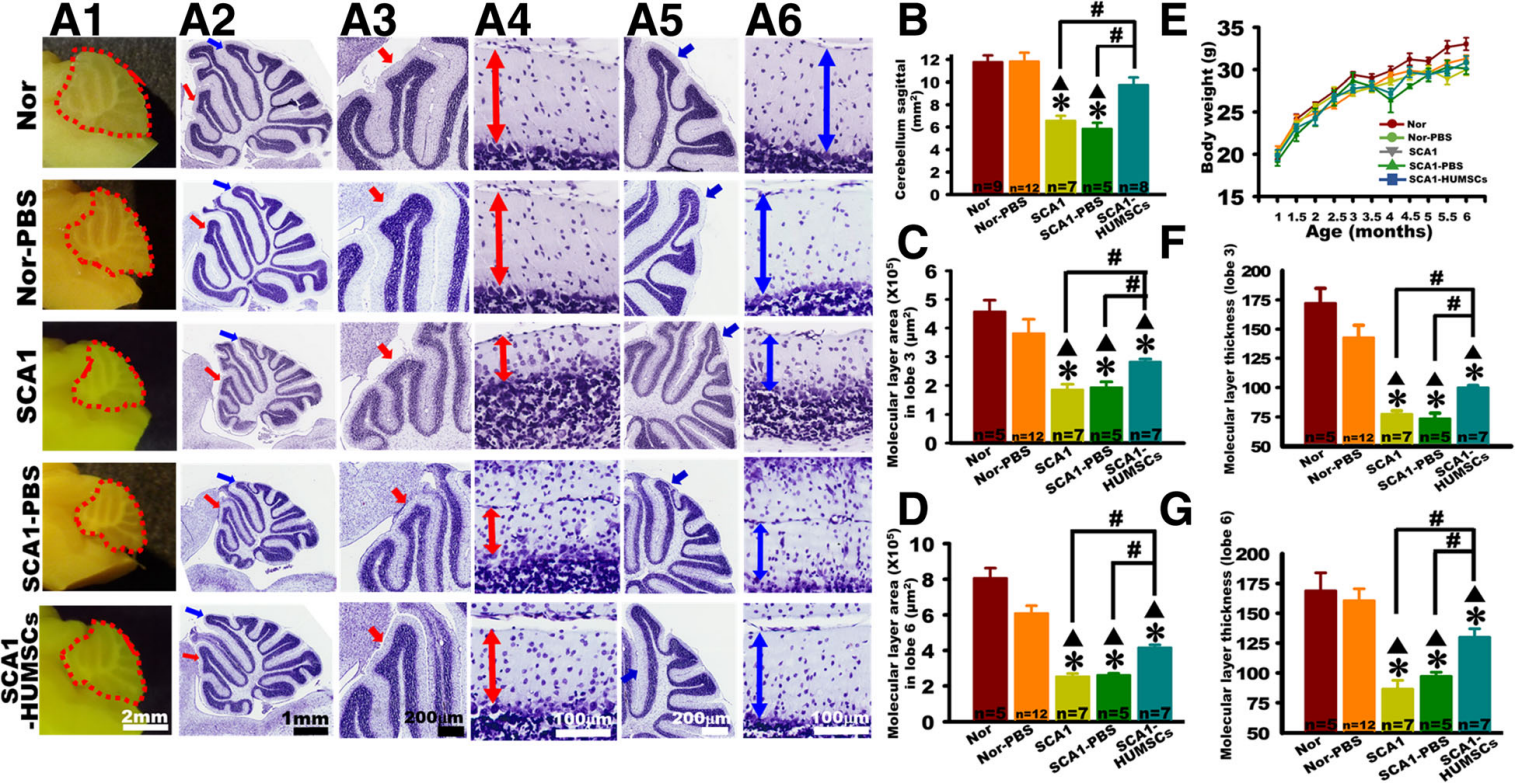
HUMSCs transplantation alleviated molecular layer atrophy in six-month-old SCA1 mice. Six-month-old mice were sacrificed to examine alterations within cerebellar sagittal sections (**a1**). The areas of cerebellar sagittal section were quantified. HUMSCs transplantation reduced the shrinkage of cerebellum in SCA1 mice (**b**). All groups were examined, photomicrographs of entire sagittal sections were stained with Cresyl violet (**a2**-**a6**), lobule III at low (red arrow, **a2** and **a3**) and high (**a4**) magnification, and lobule VI at low (blue arrow, **a2** and **a5**) and high (**a6**) magnification were shown. In lobules III and VI, significant reductions were found in the molecular layer areas (**c** and **d**) and thickness (**f** and **g**) in the six-month-old SCA1 and SCA1-PBS groups. HUMSCs transplants could partially mitigate these reductions in SCA1-HUMSCs mice. There were no significant differences in the body weight among mice in all groups at the same ages (**e**). * *p* < 0.05 compared with the Normal group. ▲*p* < 0.05 compared with the Normal-PBS group. # *p* < 0.05 compared with the SCA1-HUMSCs group

Cerebellar slices of each group of mice at 6 months of age were stained with Cresyl violet and observed at low and high magnifications under optical microscope (Fig. 3a1-a6). Morphological examination of sagittal cerebellar sections of six-month-old mice in the SCA1 and SCA1-PBS groups, in the more advanced stage of the disease, showed a prominent atrophy in sagittal area as compared to the other groups. HUMSCs transplantation brought significant improvement (Fig. 3a1- a2 and b). At high magnifications, the thickness and areas of molecular layer in lobules III and VI significantly decreased in the SCA1 and SCA1-PBS groups as compared to the Normal and Normal-PBS groups, indicating the disease progression in the SCA1 mice (Fig. 3a3- a6, c-d and f- g). Although not achieving complete recovery, HUMSCs transplantation nonetheless significantly ameliorated the atrophy as revealed by the increase in area and thickness of lobules III and VI (Fig. 3a3- a6, c-d and f- g).

In order to investigate whether SCA1 or transplantation of HUMSCs might affect growth, the mice was weighed every half a month. The results indicated that the average body weight increased significantly throughout the 6 months following birth with no significant differences in all the groups at the same ages (Fig. 3e).

### HUMSCs transplantation improved SCA1-induced Purkinje cell loss and atrophy

**Fig. 4:**
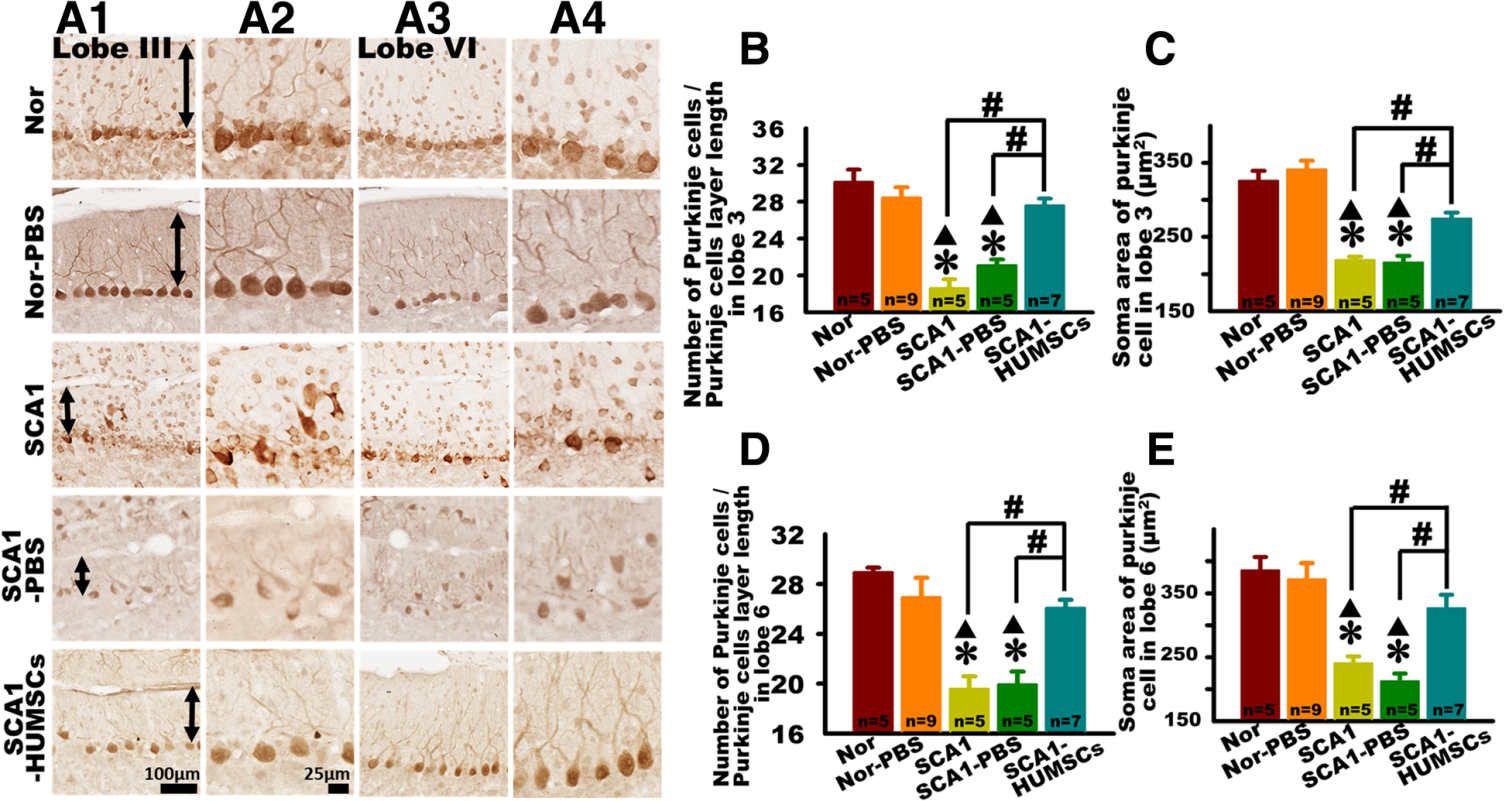
HUMSCs transplantation alleviated Purkinje cell damage SCA1 mice. Damages to the Purkinje cells in the SCA1 mice were assessed by sacrificing six-month-old SCA1 mice and observing, under optical microscope, Purkinje cells in lobules III and VI in tissue slices following immunohistochemical staining with anti-calbindin antibodies**.** Presented are representative photomicrographs of tissue slices of lobules III (**a1** and **a2**) and VI (**a3** and **a4**) in mice at 6 months of age in all groups. Purkinje cell number per unit length (**b** and **d**) and cell body area (**c** and **e**) in lobules III and VI were quantified. At 6 months of age, significant decreases were observed in both cell number and cell body area in the groups of SCA1 and SCA1-PBS. Transplantation of HUMSCs salvaged such losses (**b**-**e**). * *p* < 0.05 compared with the Normal group. ▲ *p* < 0.05 compared with the Normal-PBS group. # *p* < 0.05 compared with the SCA1-HUMSCs group

Damage to the Purkinje cells was assessed by observation of the general cellular organization, cell density and cell soma area. The largest of the obtained sagittal cerebellar slice with the best integrity in lobules III and VI were chosen for examinations following immunohistochemical staining using anti-calbindin antibodies (Fig. 4a1-a4).

In both lobules III and VI of the six-month-old SCA1 and SCA1-PBS mice, a relatively disorganized cluster of Purkinje cells and lower number of Purkinje cells per unit length were observed as compared to those in the Normal and Normal-PBS mice (Fig. 4a1- a4, Additional file 3: Figure S3). Moreover, the Purkinje cell body areas of Lobules III and VI in the SCA1 and SCA1-PBS mice were smaller than those in the Normal and Normal-PBS groups (Fig. 4a1- a4, b- e). The number and the soma area of Purkinje cells in both Lobules III and VI markedly recovered in the SCA1-HUMSCs group as compared with the SCA1 and SCA1-PBS groups (Fig. 4a1- a4, b- e). HUMSCs transplantation seemed to ameliorate Purkinje cell losses and atrophy.

**Fig. 5:**
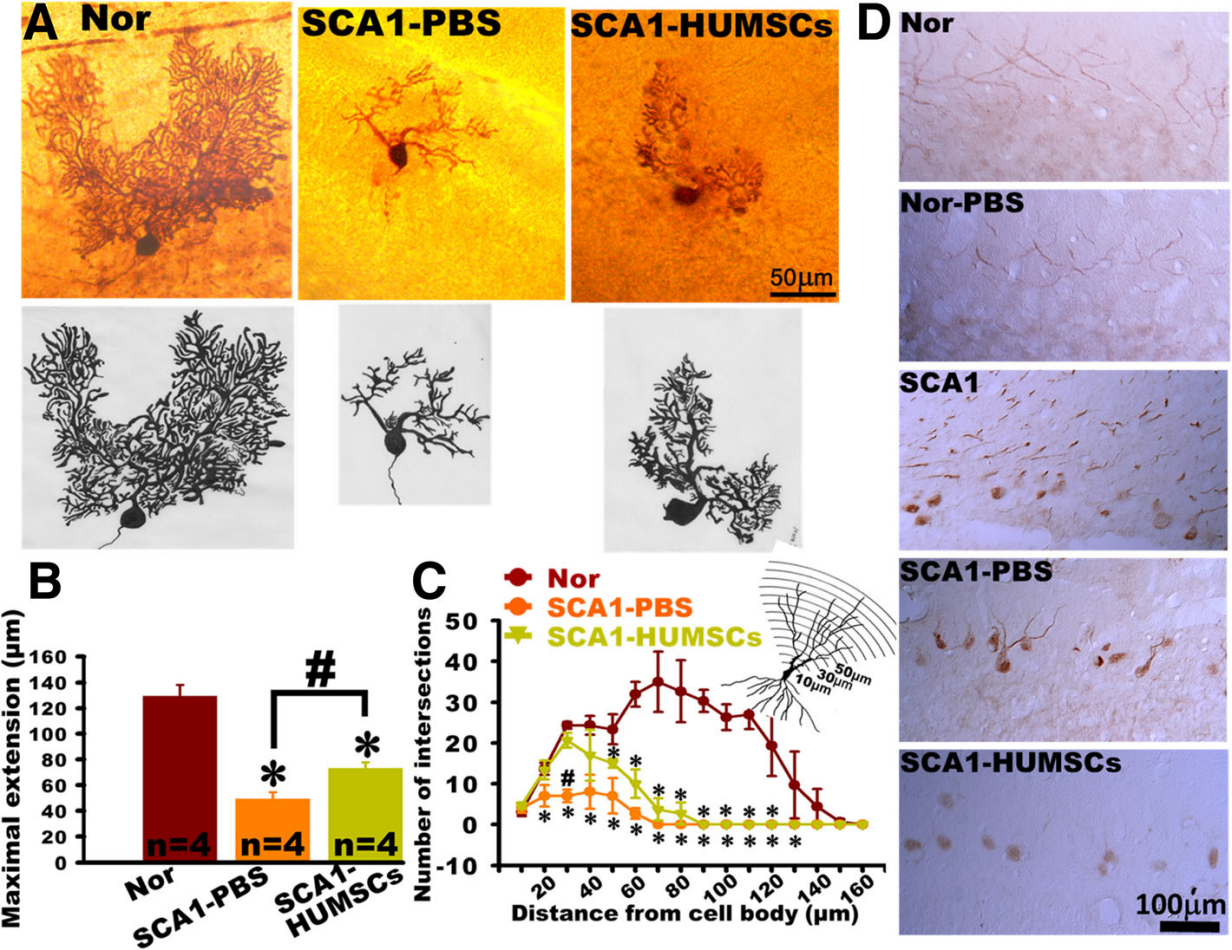
HUMSCs transplantation alleviated decline in dendritic branching in Purkinje cells. Cerebellar atrophy, as assessed by decline in dendritic branching in the Purkinje cells, was followed by morphological observations in cerebellar slices (300 μm thick) from six-month-old mice following intracellular biotin injection and immunostaining with anti-biocytin antibodies. Presented are representative photomicrographs and corresponding schematic drawings of cerebellar slices from mice in the groups of Normal, SCA1-PBS and SCA1-HUMSCs (**a**). Centered at the soma body, concentric rings with a radial distance of 10 μm between circles were used for quantification of the number of intersections. Quantifications using Sholl’s analysis indicated that HUMSCs transplantation partially preserved Purkinje cell extensions (**b**) and ameliorated shrinkage of intersections in the proximal regions (**c**) in SCA1 mice. Cerebellar sections were stained with an ATXN1 antibody. Presence of robust staining of ATXN1 protein in nuclei were found in the groups SCA1 and SCA1-PBS mice but not in the Normal, Normal-PBS, and SCA1-HUMSCs mice (**d**). * *p* < 0.05 compared with the Normal group. # *p* < 0.05 compared with the SCA1-HUMSCs group

Dendritic atrophy of the Purkinje cells was examined in six-month-old mice using intracellular injection biocytin. In the six-month-old Normal group mice, the dendrites of the Purkinje cells had numerous branches and high complexity. In contrast, the Purkinje cell dendrites of the SCA1-PBS group appeared shriveled and had only few branches. The SCA1-HUMSCs group appeared to have higher numbers of branches and longer dendritic lengths (Fig. 5a).

The maximal sagittal extensions from the soma to the longest dendritic ends were further quantified. As shown in Fig. 5b, the maximal sagittal extension of the Normal group was 129.64 ± 8.64 μm, which decreased to 49.33 ± 5.35 μm in the SCA1-PBS group. Although still not completely recovering to the corresponding dimensions of the Normal group, the SCA1-HUMSCs mice nonetheless exhibited dendritic lengths of 73.34 ± 4.55 μm, demonstrating an attenuation of the atrophy.

Centered at the soma body, concentric rings with a radial distance of 10 μm between circles were used for quantification as shown in Fig. 5c. As compared with the Normal group, the dendritic branches of the SCA1-PBS group began to decrease at 20 μm from the soma body. In contrast, dendritic branches of the SCA1-HUMSCs mice declined significantly only at the distance of 50 μm from centroid (Fig. 5c).

Cerebellar slices were stained with ATXN1 antibody to examine the distribution and pattern of ATXN1 protein. ATXN1 protein was hardly detected in somata of Purkinje cell from the Normal and Normal-PBS groups, but was only found weakly stained in the dendrites. However, in both SCA1 and SCA1-PBS mice, ATXN1 protein was readily detected in the cell bodies and throughout the dendrites of the Purkinje cells. Moreover, it was robustly stained in the nuclei. ATXN1 protein was detectable to a lesser extent in the cell bodies but not in the dendrites of Purkinje cells of SCA1-HUMSCs mice (Fig. 5d).

### Engrafted HUMSCs survived in the cerebellum and did not differentiate into neurons or astrocytes

**Fig. 6:**
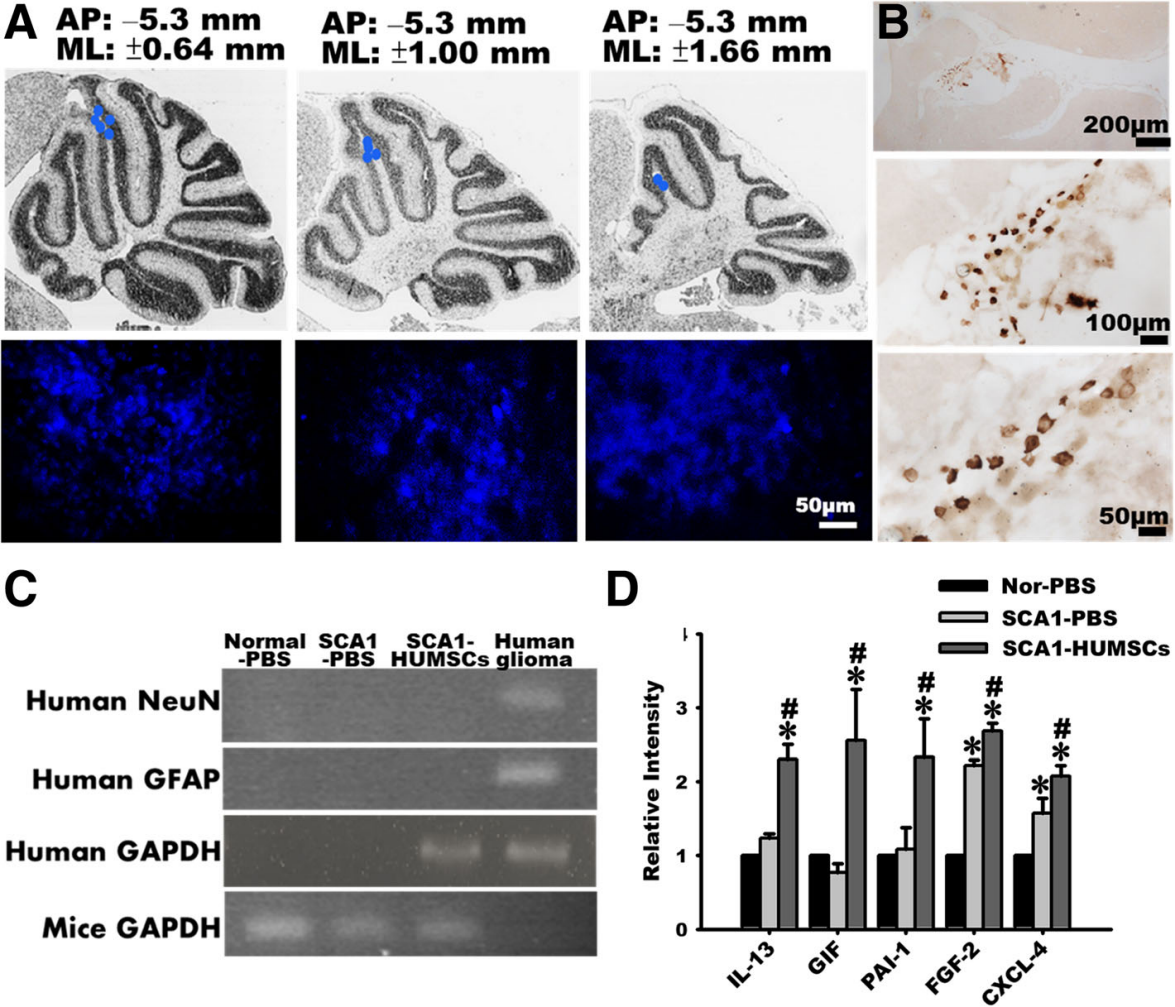
Transplanted HUMSCs survived in the grafted-cerebellum and did not differentiate into neuronal cells. HUMSCs were labeled with the blue fluorescent nuclear staining bisbenzamide 48 h prior to transplantation and their viability and distribution were followed when the mice were sacrificed at 6 months of age. Blue fluorescent cell clusters were identified at lateral levels of bregma 1.2 mm, 2.1 mm, and 3.0 mm, with upper panel displaying the relative sites, lower panel showing the fluorescence photographs (**a**). Samples from six-month-old SCA1-HUMSCs mice were immunostained with antibody against human specific nuclei antigen (**b**). **c** RT-PCR was further applied to detect human NeuN and GFAP mRNA expressions in SCA1 mice. Human NeuN and GFAP mRNAs were not detectable in the SCA1-HUMSCs group. Human glioma cells were used as a positive control of human NeuN and GFAP mRNAs assay. **d** The expression of 174 human cytokines in mouse cerebella was examined 5 months after the HUMSCs transplantation. IL-13, GIF, PAI-1, FGF-2, and CXCL-4 were significantly increased in the group of SCA1-HUMSCs. * *p <* 0.05 compared with the Normal-PBS group. # *p <* 0.05 compared with SCA1-PBS

Blue fluorescence emitting HUMSCs were observed in locations extending 1.2–3 mm from midline, with a radius about 1.8 mm in the cerebellum of SCA1 mice (Fig. 6a). Viability status of the transplanted HUMSCs was examined through immunohistochemical staining using anti-human specific nuclei antigen antibody. The results indicated that HUMSCs remained viable in the cerebellum of the six-month-old SCA1-HUMSCs group mice (Fig. 6b).

To further investigate whether HUMSCs had differentiated into neuronal cells or astrocytes in the mice cerebellum, human NeuN and GFAP were ascertained by RT-PCR. Human glioma acted as positive controls. The expression of human GAPDH was detected in the human glioma and SCA1 + HUMSCs group. However, neither human NeuN nor GFAP mRNAs was detectable in the cerebella of the Normal-PBS, SCA1-PBS and SCA1-HUMSCs groups, indicating that the HUMSCs did not differentiate into neurons or astrocytes (Fig. 6c).

To further elucidate the underlying mechanisms of the beneficial effects of HUMSC transplantation, cytokine expressions in the cerebellar tissues were examined using human cytokine arrays. The results showed that significantly elevated expressions of several growth promoting factors were associated with HUMSCs transplantation. These growth-promoting factors included IL-13, GIF, PAI-1, FGF-2 and CXCL-4 (Fig. 6d and Additional file 4: Figure S4).

## Discussion

### Pathological changes in the SCA1 mice

Based on the results of the rotarod test, previous studies reported that decrease in motor functions in SCA1 transgenic mice starting at the age of 5 weeks and lasting till 18 weeks [31, 32]. Furthermore, shrinkage of the molecular layer and loss of Purkinje cells began at 10 weeks of age and dropped significantly at 18 or 23 weeks of age [31–33]. Similarly, results from the rotarod, balance beam test and open field test in our study indicated that one- to six-month-old SCA1 mice exhibited deteriorating motor functions. Morphological results revealed that cerebellar degeneration was noticeable at 6 months of age. Significant reduction in the thickness and area of the molecular layer, number of Purkinje cells, and soma area of Purkinje cells were observed.

In this study, amplitudes of compound motor action potentials of the lumbar spinal nerve decreased markedly in the six-month-old SCA1 mice. It was most likely that, following electrical stimulation, fewer muscle fibers were elicited to contract in the SCA1 group which could be attributed to the decline of spinal motor neurons governing gastrocnemius muscle contraction. We suggest that the output of Purkinje cells to the cerebellar deep nuclei might diminish as a result of the damaged and death of Purkinje cell. Subsequently, the efferent of cerebellar deep nuclei might influence ventral lateral nucleus of thalamus, motor cortex and spinal cord. Eventually, it might affect the innervation from the spinal cord to the gastrocnemius muscle at the age of 6 months. Since there were no statistical differences in the conduction latency or duration of compound motor action potentials, it is possible that myelination of the peripheral nerves had remained intact. Intriguingly, in a SCA1-knock-in mouse model, Takechi et al. found lower amplitudes of hind limb muscle action potentials, delayed nerve conduction and prolonged latency and duration of compound motor action potentials [28]. Different genetic background of the mice might have contributed to the discrepancies in the electrophysiological studies.

### HUMSCs transplantation effectively ameliorated pathological alterations in the SCA1 mice

Our study indicated that transplantation of 10^6^ HUMSCs into the cerebellum of SCA1 mice could effectively improve motor performance of the SCA1 mice as evaluated by the open field test, rotarod and balance beam tests. Cresyl staining revealed that HUMSCs transplantation efficiently reduced thinning of the molecular layer in the cerebellum. In addition, results from immunocytochemical staining using anti-calbindin antibody indicated implantation of HUMSCs alleviated Purkinje cell loss in six-month-old SCA1 mice (*p* < 0.05). When Purkinje cells were injected and labeled with biocytin, we found that HUMSCs transplants substantially decreased Purkinje cell degeneration in the SCA1 mice (*p* < 0.05).

In the present study, the SCA1-HUMSCs group did not display improved motor function until 3to 4 months of age when comparing to the SCA1 and SCA1-PBS groups, indicating that HUMSCs transplants require 2 to 3 months to ameliorate pathological microenvironment and demonstrate their therapeutic effects.

Chintawar and colleagues transplanted mice neural precursor cells (NPCs) into the cerebella of SCA1 mice at 24 weeks of age [29]. The results, as assessed by the rotarod test, showed that NPCs implantation prolonged the latency to fall 1 month after transplantation. Two months after grafting, the grip strength of the SCA1 mice increased significantly. In addition, NPC grafts reduced shrinkage of Purkinje cells in the SCA1 mice [29]. In agreement with our finding, Matsuura et al. demonstrated that intrathecal injection of mouse bone marrow mesenchymal stem cells into SCA1 mice mitigated deficits in motor coordination and suppressed cerebellar atrophy [33]. However, technical and ethical difficulties in obtaining sufficient and appropriate human NPCs and bone marrow mesenchymal stem cells (BM-MSCs) have limited the application of these therapies. The advantages of the HUMSCs versus the NPCs and mouse BM-MSCs include that they are human cells with potential as a therapy for a human disease, are readily available and do not require manipulation/differentiation prior to administration. In addition, we have found that HUMSCs possess the ability for long-term survival in different organs after xeno-transplantation, without the need for immunological suppression, suggesting that HUMSCs might be a good stem cell source for transplantation for clinical medicine [15–24].

### The underlying mechanisms of reduced cerebellar degeneration in the SCA1 mice by HUMSCs transplantation

Based on the data obtained using RT-PCR in our study, it is supposed that HUMSCs did not directly differentiate into neurons or astrocytes after transplantation into the cerebella of the SCA1 mice, but rather released certain nourishing factors to enhance the endogenous mechanisms of tissue repair. Interestingly, HUMSCs express different cytokines when engrafted into the fibrotic liver, ischemic cortex and injured spinal cord, depending on respective pathological microenvironments [18, 19, 22]. As demonstrated in our previous study, transplantation of HUMSCs could serve as a therapeutic option for ischemic stroke [19]. Implanted HUMSCs released neuroprotective and growth-associated cytokines, including neutrophil-activating protein-2 (NAP-2), angiopoientin-2, brain-derived neurotrophic factor, CXCL-16 and platelet-derived growth factor-AA. These cytokines could promote endogenous neurogenesis, protect neurons from damage, improve motor function after ischemic stroke, and therefore contribute to the overall therapeutic effects [19] At the same time, when HUMSCs are implanted into the rat spinal cord after complete transection, instead of differentiating into neurons, astrocytes, or oligodendrocytes, they release human cytokines and growth factors, such as neurotrophin-3, NAP-2, basic fibroblast growth factor, glucocorticoid induced tumor necrosis factor receptor family, and vascular endothelial growth factor receptor 3, which may be involved in the repair and regeneration of the spinal cord [18]. Furthermore, without differentiating into hepatocytes, HUMSCs grafted into rat fibrotic liver release considerable amount of human prolactin, leukemia inhibitory factor, and cutaneous T cell-attracting chemokine [22]. Therefore, it is likely that HUMSCs may release different cytokines in response to the pathophysiological microenvironments accordingly. This speculation is supported by our findings that five kinds of growth-associated human cytokines increases in the group of SCA1-HUMSCs. As revealed by our human cytokine assay, HUMSCs grafted in the cerebellum of SCA1 mice released interleukin 13 (IL-13), glucocorticoid-increasing factor (GIF), plasminogen activator inhibitor-1 (PAI-1), basic fibroblast growth factor (FGF-2) and CXC chemokine ligand 4 (CXCL-4). In the HUMSC-grafted cerebellum, the up-regulation of human IL-13、GIF and PAI-1 may be related to anti-inflammation [34–36]**.** An increased expression of IL-13 is associated with enhanced MSCs migration and functionality [36]**.** FGF-2 is upregulated in the damage cerebellum to initiate neovascularization and facilitate neurogenesis [37]**.** Likewise, CXCL-14 may stimulate the proliferation, chemotaxis, and tube formation of endothelial cells and are essential for the development of blood vessels [38]**.** We speculate that some other cytokines and growth factors beyond the 174 that we assessed may also protect SCA1 cerebella. Interestingly, human FGF-2 and CXCL-4 in the SCA1-PBS group (which no received HUMSCs implantation) were higher than those in the Nor-PBS group. We speculate that the specificities of human FGF-2 or CXCL-4 antibodies were cross-reacted to the mouse FGF-2 and CXCL-4, representing that mouse FGF-2 and CXCL-4 increased in the degenerative cerebella. The significant raises of mouse FGF-2 and CXCL-4 in the SCA1-PBS group may be a self-compensation to trigger recovery, eventually, the kind and quantity of cytokine released from SCA1 mice might be insufficient to repair damaged cerebella. In the near future, we will treat SCA1 mice with one or a combination of those cytokines to investigate whether we see the same beneficial effects as when transplanting HUMSCs.

## Conclusions

The HUMSCs transplanted into the cerebella of SCA1 transgenic mice significantly ameliorated motor symptoms and cerebellar degeneration. The rescue, even if it is only partial, of such a severe disease phenotype like SCA1 is an important finding. The present study provides evidence to support the therapeutic potential of HUMSCs in patients suffering from SCA1.

## Additional files

Additional file 1: **Figure S1.** Chromosomal karyotyping and surface markers of HUMSCs in vitro. To analyze the copy number of 23 chromosomes of HUMSCs in vitro (passage 10th), the CytoScan 750 K Array (Affymetrix) was used to screen the chromosomal karyotype. The X-axis represents the chromosome number (No. 1–22, XX), the right Y-axis represents the copy number of chromosome (0, 1, 2, or 3), and the blue line is the copy number of chromosomes performed by the company of Genetics Generation Advancement. The result indicated that the chromosomes No. 1–22 and sex chromosome (X) are all two sets. (B) Flow cytometry analyses of surface markers of HUMSCs in vitro. HUMSCs were cultured for 10 passages and then labeled with CD44, CD105 and HLA-DR antibodies. White areas represent negative controls and red areas represent the specific binding for indicated antigens. The results revealed that HUMSCs transplanted into mice were positive for CD44 and CD105 but negative for HLA-DR. (PDF 142 kb)

Additional file 2: **Figure S2.** Quantitative method of Purkinje cell number in the Lobules III and VI. The cerebellar slices of all groups were immunostained with anti-calbindin to label the Purkinje cell. Quantitative analysis of Purkinje cell number was made according to numbers of Purkinje cell (red) in the unit length of Purkinje cell layer (green line) in Lobules III and VI. (PDF 100 kb)

Additional file 3: **Figure S3.** Low-magnification images show the anti-calbindin immunostaining for Purkinje cells in cerebellum. The cerebellar slices of all groups were immunostained with anti-calbindin to label Purkinje cells in cerebellum (Column A for Normal group, B for Normal-PBS group, C for SCA1 group, D for SCA1-PBS group, and E for SAC1-HUMSCs group). The lower two panels are magnified images for Lobules III (red arrows) and VI (blue arrows), respectively, in the top panels. The results demonstrated that Purkinje cells were disorganized in alignment and sparse in quantity in Lobules III and VI of the six-month-old SCA1 and SCA1-PBS mice. (PDF 304 kb)

Additional file 4: **Figure S4.** Human cytokine antibody array of the mouse cerebella. One hundred and seventy-four human cytokines were blotted onto the membranes and their corresponding positions are shown as in the right panels. Five growth-promoting human cytokines were significantly increased in the SCA1-HUMSCs group. (PDF 179 kb)

## Data Availability

The dataset will be publically released upon acceptance of this manuscript, but are currently available for reviewer access.

## Abbreviations

BM-MSCs: Bone marrow mesenchymal stem cells
DMEM: Dulbecco’s modified Eagle’s medium
FBS: Fetal bovine serum
GAPDH: Glyceraldehyde 3-phosphate dehydrogenase
GFAP: Glial fibrillary acidic protein
HUMSCs: Human umbilical mesenchymal stem cells
NAP-2: Neutrophil-activating protein-2
NPCs: Neural precursor cells
PB: Phosphate buffer
PBS: Phosphate buffered saline
SCA1: Spinocerebellar ataxia type 1
