# Supplementary figures and images for: Xenografting of human umbilical mesenchymal stem cells from Wharton’s jelly ameliorates mouse spinocerebellar ataxia type 1
Source: Transl Neurodegener. 2019 Sep 5;8:29. doi: 10.1186/s40035-019-0166-8 (PMC6727337; doi:10.1186/s40035-019-0166-8)

# Supplemental Figure 1

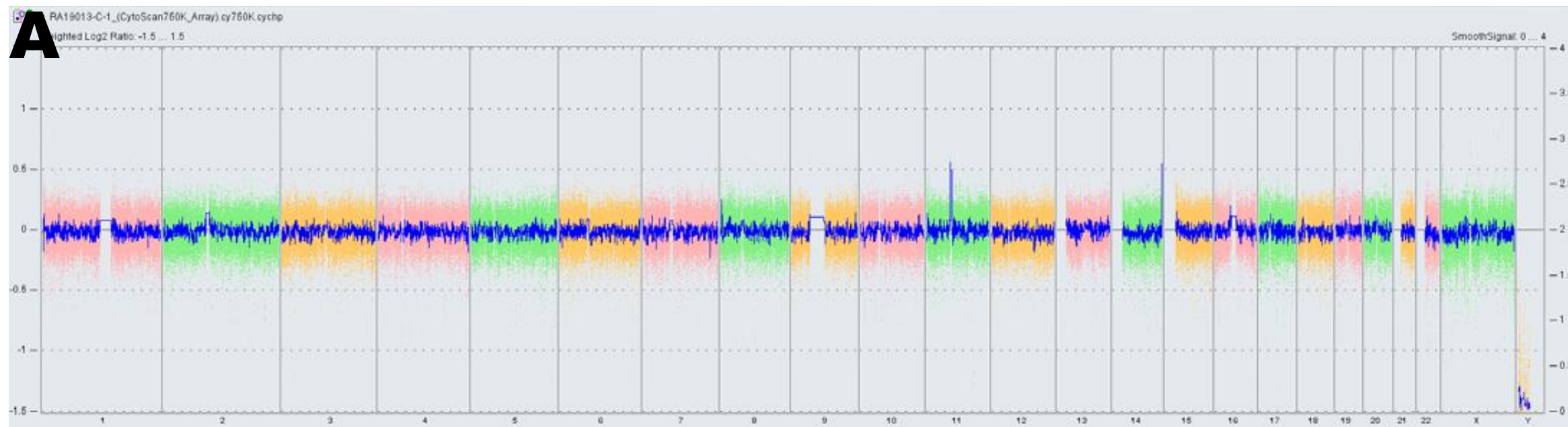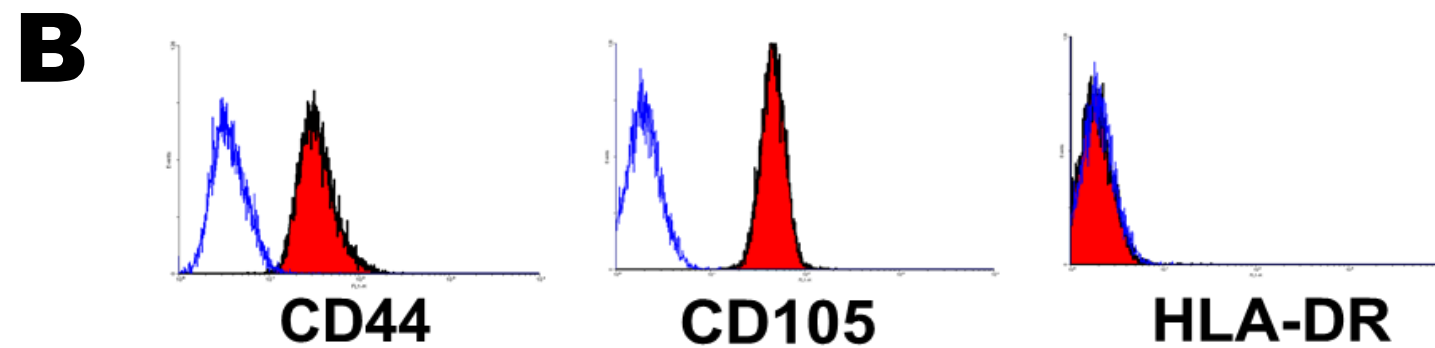

Supplement: Supplementary file 4 — Figure S1. Chromosomal karyotyping and surface markers of HUMSCs in vitro. To analyze the copy number of 23 chromosomes of HUMSCs in vitro (passage 10th), the CytoScan 750 K Array (Affymetrix) was used to screen the chromosomal karyotype. The X-axis represents the chromosome number (No. 1–22, XX), the right Y-axis represents the copy number of chromosome (0, 1, 2, or 3), and the blue line is the copy number of chromosomes performed by the company of Genetics Generation Advancement. The result indicated that the chromosomes No. 1–22 and sex chromosome (X) are all two sets. (B) Flow cytometry analyses of surface markers of HUMSCs in vitro. HUMSCs were cultured for 10 passages and then labeled with CD44, CD105 and HLA-DR antibodies. White areas represent negative controls and red areas represent the specific binding for indicated antigens. The results revealed that HUMSCs transplanted into mice were positive for CD44 and CD105 but negative for HLA-DR. (PDF 142 kb) [file 40035_2019_166_MOESM1_ESM.pdf]

**Supplemental Figure 3**

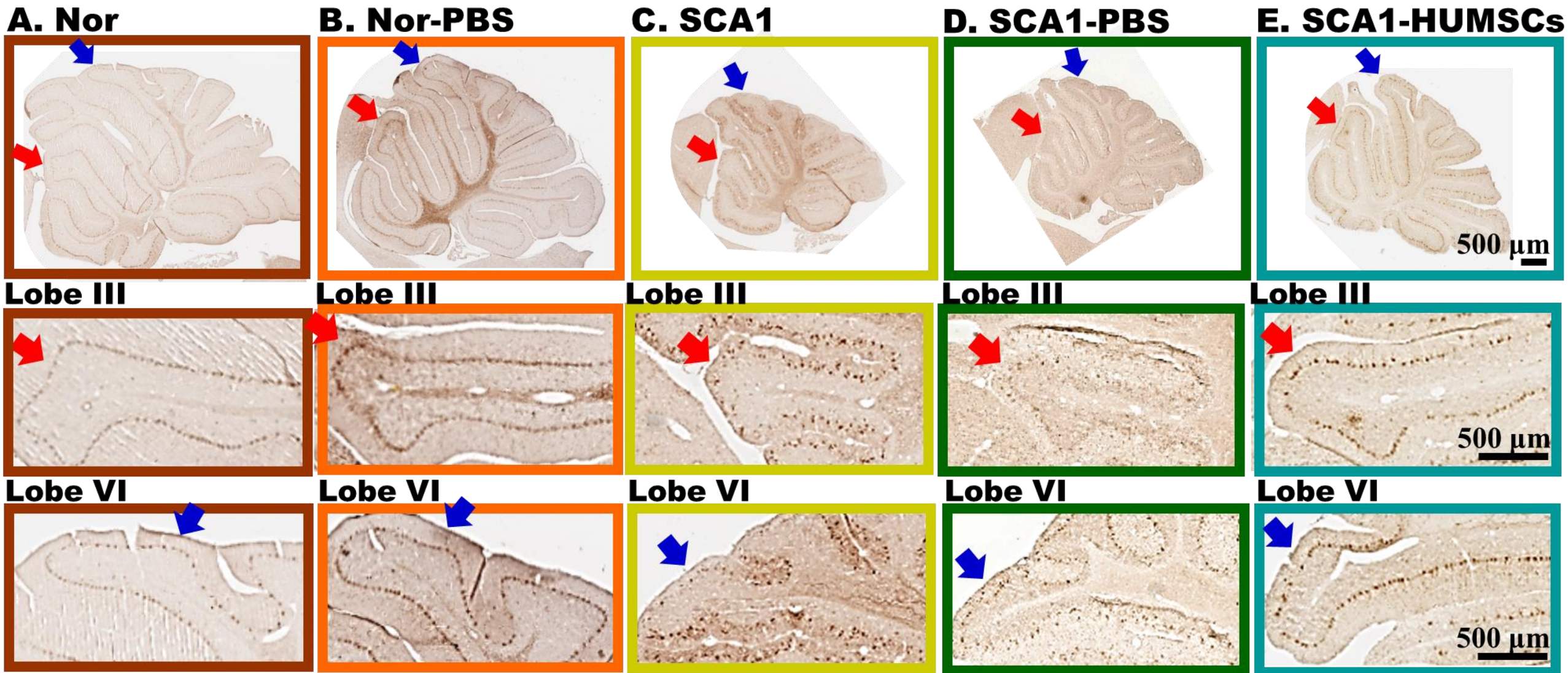

Supplement: Supplementary file 6 — Figure S3. Low-magnification images show the anti-calbindin immunostaining for Purkinje cells in cerebellum. The cerebellar slices of all groups were immunostained with anti-calbindin to label Purkinje cells in cerebellum (Column A for Normal group, B for Normal-PBS group, C for SCA1 group, D for SCA1-PBS group, and E for SAC1-HUMSCs group). The lower two panels are magnified images for Lobules III (red arrows) and VI (blue arrows), respectively, in the top panels. The results demonstrated that Purkinje cells were disorganized in alignment and sparse in quantity in Lobules III and VI of the six-month-old SCA1 and SCA1-PBS mice. (PDF 304 kb) [file 40035_2019_166_MOESM3_ESM.pdf]
