# Supplementary material for: Xenografting of human umbilical mesenchymal stem cells from Wharton’s jelly ameliorates mouse spinocerebellar ataxia type 1
Source: Transl Neurodegener. 2019 Sep 5;8:29. doi: 10.1186/s40035-019-0166-8 (PMC6727337; doi:10.1186/s40035-019-0166-8)
Supplement: Supplementary file 5 — Figure S2. Quantitative method of Purkinje cell number in the Lobules III and VI. The cerebellar slices of all groups were immunostained with anti-calbindin to label the Purkinje cell. Quantitative analysis of Purkinje cell number was made according to numbers of Purkinje cell (red) in the unit length of Purkinje cell layer (green line) in Lobules III and VI. (PDF 100 kb) [file 40035_2019_166_MOESM2_ESM.pdf]

Supplemental Figure 2

➢ **The method of purkinje cells counting using anti-calbindin immunostaining**

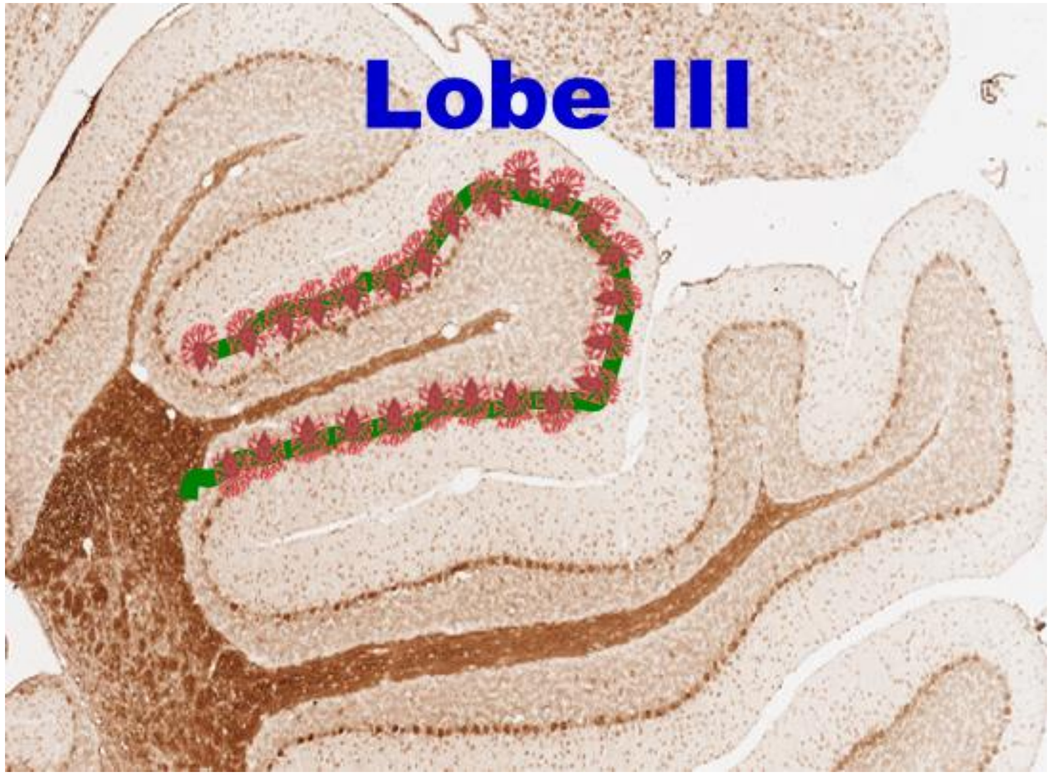

$$\frac{\text{The total number of purkinje cell in Lobe III}}{\text{Total length of purkinje cell layer in Lobe III (the length of the green line)}}$$
